# Therapeutic Discrimination

**Published:** 1910-04-23

**Authors:** 


### The Hospital

A Journal of

The Medical Sciences and Hospital Administration.

New Series. No. 166, Vol. VII. [No. 1233, Vol. XLVIII] SATURDAY, APRIL 23, 1910.

### Therapeutic Discrimination.

A somewhat heated, but very important and instructive, controversy which has been lately carried on in the correspondence columns of the *British Medical Journal* illustrates very clearly the need for that scientific scepticism of which Professor Huxley was so insistent an advocate. The profession, in fact, like humanity at large, is much too apt to accept reiteration for truth, heedless of the patent self-interest of those who do the reiterating. Advertisers of new remedies, or of old remedies under new names, or of rubbish which is not fit to have a name at all, have only to cry their wares loudly enough, to extol their alleged virtues often enough, and the profession too frequently responds with a childish faith which does little credit to the thoroughness of the medical student's scientific training. That the laity, and especially the uneducated sections of it, should confound assertion with proof is not astonishing; human nature is so constituted. But that the members of the most scientific profession should do the same is unpardonable, and ought to be impossible.

No better instance of this unfortunate tendency can be selected than the subject of the discussion to which allusion has been made—to wit, the virtues and the limitations of treatment by the organic arsenic compounds collectively known as the arylarsonates. The original arylarsonate with which experiments were made was the property—unless we are in error—of a foreign firm, and was sold under the trade-name of atoxyl. This drug was tried, somewhat empirically, for sleeping sickness, in the hope no doubt that the contained arsenic might poison the trypanosomes in the circulation without also poisoning the individual. At first some results were obtained which, whether or not due to the atoxyl, were regarded as highly encouraging, and hundreds, if not thousands, of sufferers were treated by this method for prolonged periods of time. Yet in the end disappointment ensued, and only lately an authority of eminence has declared that no case of sleeping sickness has been cured by the drug. In the "boom" which followed this extensive use of atoxyl, other arylarsonates were marketed by various firms of manufacturing druggists, British and foreign, and their sale pushed, under trade-names, by all the resources of the modern advertisement manager. Not only sleeping sickness, remedies for which are not likely ever to command much sale in Britain, but other infective diseases known or suspected to be due to organisms even remotely similar to trypanosomes, and also blood diseases for which arsenic has sometimes been found of value, were treated with these drugs by enterprising and enthusiastic researchers; and their published works on this form of treatment were quoted far and wide by those interested in the sale of arylarsonates. Not contented with those diseases, such as sleeping sickness, for which no known cure exists, and therefore any experiment in therapeutics is justifiable, the forward school applied these powerfully toxic drugs to other diseases, and especially to syphilis. Why this disease should have been thus selected is difficult to say, for there are indeed few serious disorders for which medical treatment is so thoroughly satisfactory when conscientiously carried out. At any rate, it was so selected, and many clinicians with ample opportunities for testing new remedies for syphilis gave the arylarsonates a trial. With few exceptions they reported only disappointment, and on almost all sides the result of the experiment has been a rapid return to the old mercurial regimen in one or other of its forms.

So far, so good; a new method has been on trial and has failed. That is nothing very novel, and only by such trial can advances ever be made. In passing it may be remarked that a method which is solely empirical needs abandonment at once when it fails; whereas it is legitimate with one which has a rational basis to endeavour by many improvements to obtain ultimate success. But beyond failure in promoting cure it soon became obvious that danger attends the prolonged administration of arylarsonates. Blindness from double optic atrophy has been reported so many times that even were the treatment of proved value its disadvantages would have to be very carefully weighed. Here, then, the profession has the opportunity for the exercise of therapeutic discrimination. On the one side are the makers of various arylarsonates, filling the advertisement columns of the medical press with the praises of their products, often by means of garbled or obsolete extracts from the writings of hyperenthusiastic pioneers in their usage; on the other is the steadily growing impression among those best qualified to judge that for syphilis at least the use of these compounds is unnecessary, impolitic, and unjustifiable. Unfortunately, for every original paper in this latter sense that the ordinary practitioner sees, he has thrust upon his notice ten or twenty or a hundred of the misleading advertisements in question; and there is grave danger that he may mistake *ex parte* statement for argument, and repetition for scientific proof. This, of course, should not be; that it is so is a reproach to medical education. But since we live in an imperfect world, and since the tendency to accept the not always quite ingenuous statements of the commercial traveller or the commercial pamphlet is one whose existence in the profession has to be recognised, however much it may be deplored, the study of the correspondence columns of the *British Medical Journal* for the month of March is an exercise which can be recommended very strongly to all those who are thinking of buying arylarsonates or other much advertised proprietary medicines for the treatment of their patients.

